# H_2_S in Horticultural Plants: Endogenous Detection by an Electrochemical Sensor, Emission by a Gas Detector, and Its Correlation with L-Cysteine Desulfhydrase (LCD) Activity

**DOI:** 10.3390/ijms23105648

**Published:** 2022-05-18

**Authors:** María A. Muñoz-Vargas, Salvador González-Gordo, José M. Palma, Francisco J. Corpas

**Affiliations:** Group of Antioxidants, Free Radicals and Nitric Oxide in Biotechnology, Food and Agriculture, Department of Biochemistry, Cell and Molecular Biology of Plants, Estación Experimental del Zaidín, Spanish National Research Council (CSIC), C/Profesor Albareda 1, E-18008 Granada, Spain; mangeles.munoz@eez.csic.es (M.A.M.-V.); salvador.gonzalez@eez.csic.es (S.G.-G.); josemanuel.palma@eez.csic.es (J.M.P.)

**Keywords:** Allium, hydrogen sulfide, garlic, gas detector, ion-selective microelectrode, L-cysteine desulfhydrase, isozymes

## Abstract

H_2_S has acquired great attention in plant research because it has signaling functions under physiological and stress conditions. However, the direct detection of endogenous H_2_S and its potential emission is still a challenge in higher plants. In order to achieve a comparative analysis of the content of H_2_S among different plants with agronomical and nutritional interest including pepper fruits, broccoli, ginger, and different members of the genus Allium such as garlic, leek, Welsh and purple onion, the endogenous H_2_S and its emission was determined using an ion-selective microelectrode and a specific gas detector, respectively. The data show that endogenous H_2_S content range from pmol to μmol H_2_S · g^−1^ fresh weight whereas the H_2_S emission of fresh-cut vegetables was only detected in the different species of the genus Allium with a maximum of 9 ppm in garlic cloves. Additionally, the activity and isozymes of the L-cysteine desulfhydrase (LCD) were analyzed, which is one of the main enzymatic sources of H_2_S, where the different species of the genus Allium showed the highest activities. Using non-denaturing gel electrophoresis, the data indicated the presence of up to nine different LCD isozymes from one in ginger to four in onion, leek, and broccoli. In summary, the data indicate a correlation between higher LCD activity with the endogenous H_2_S content and its emission in the analyzed horticultural species. Furthermore, the high content of endogenous H_2_S in the Allium species supports the recognized benefits for human health, which are associated with its consumption.

## 1. Introduction

H_2_S is a key signaling molecule that plays multiple functions in many physiological and pathological processes in humans, regulating the basal metabolism, central nervous system, blood pressure, gastrointestinal motility, inflammation, the immune system, or cancer, among others [1,2,3,4,5,6,7]. In higher plants, H_2_S also has relevant functions due to its direct or indirect implication in physiological functions including seed germination, root development, plant growth, stomatal closure, senescence, and fruit ripening as well as in the mechanism of response against adverse environmental conditions [8,9,10,11,12,13]. H_2_S is part of the sulfur metabolism being enzymatically generated by different enzymes present in diverse subcellular compartments including cytosol, plastids, and mitochondria [14]. At the biochemical level, H_2_S mediates the regulation of protein function by a posttranslational modification designated persufidation which involved the thiol group of cysteine residues from target proteins [15,16,17]. However, the detection of endogenous hydrogen sulfide and its possible emission continues to be a scientific challenge due to the complex biochemistry that is affected by its interaction with peptides and proteins, pH, cellular location, type of biological samples, etc. Different methodologies allow the detection of endogenous H_2_S in different types of biological samples such as high-performance liquid chromatography (HPLC), gas chromatography (GC), colorimetric, specific fluorescence probes, ion-selective electrode (ISE), amperometric (polarographic) H_2_S sensor, ozone-based chemiluminescence detection among others and all have advantages and disadvantages as well as different detection limits [18,19,20,21]. 

L-Cysteine desulfhydrase (LCD, EC 4.4.1.28) is considered one of the main cytosolic enzymatic sources of H_2_S in Arabidopsis cells which is also generated by other enzymes such as the chloroplastic sulfite reductase (SiR, EC 1.8.7.1) or the mitochondrial bifunctional D-cysteine desulfhydrase/1-aminocyclopropane-1-carboxylate deaminase (DCDES1, EC 4.4.1.15) and D-cysteine desulfhydrase 2 (DCDES2, EC 4.4.1.15) [14,22,23]. The LCD catalyzes the following reaction: L-cysteine + H_2_O → pyruvate + NH^4+^ + H_2_S + H^+^, and it requires pyridoxal 5′-phosphate (PLP) as cofactor [23,24]. This enzyme is involved in diverse processes such as root development [25], stomatal closure [26,27,28], leaf senescence [29], fruit ripening [30,31], and response to diverse stresses [32].

Aiming to correlate the potential relationship between endogenous H_2_S, its potential emission, and the activity of the LCD, the present study provides a comparative analysis of these parameters in some horticultural plants such as pepper fruits, broccoli, ginger, fennel, eggplant, leek, garlic, Welsh and onion, which are relevant to human nutrition. The Allium species, particularly garlic cloves, are horticultural plants that present the highest values of H_2_S that is well correlated with its very active metabolism of organosulfur compounds such as allicin, allyl sulfides, allyl thiosulfinate, ajoene, and S-allyl cysteine among others.

## 2. Results

**Figure 1:**
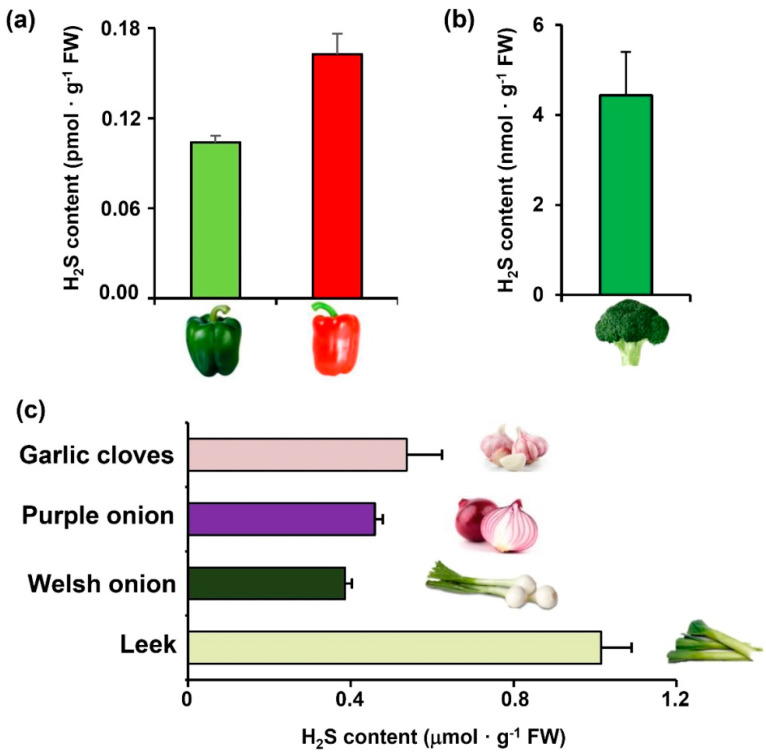
Endogenous H_2_S detection in different plant species including (**a**) sweet pepper (*Capsicum annuum* L.) fruit at distinct ripening stages (fully green and fully red), (**b**) Broccoli (*Brassica oleracea* var. Itálica), (**c**) Allium species including garlic (*Allium sativum* L.) cloves, leek (*Allium ampeloprasum* var. porrum), welsh onion (*Allium fistulosum*), and purple onion (*Allium cepa*). H_2_S was detected using a micro sulfide ion electrode.

Figure 1a–c illustrates the endogenous H_2_S content in different horticultural plant species which was measured using an ion-selective microelectrode (Arrow H_2_S™) H_2_S measurement system. Accordingly, three well-differentiated data groups can be distinguished, while the species of the Allium genus give H_2_S values in the range of μmol H_2_S · g^−1^ FW, the leek with 1 μmol H_2_S · g^−1^ FW being the species with the highest content, followed by garlic cloves with 0.54 μmol H_2_S · g^−1^ FW. Other groups are in the range of nmol H_2_S · g^−1^ FW such as broccoli but also in the range of pmol H_2_S · g^−1^ FW such as pepper fruit at different stages of ripening green and red. On the other hand, in fennel and eggplant samples the H_2_S detected was even lower, so they were not used for subsequent studies.

**Figure 2:**
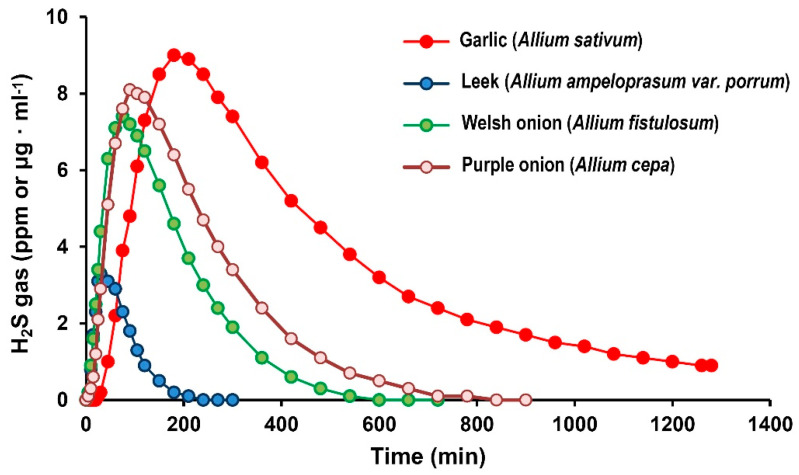
H_2_S gas emission in different plant species including garlic (*Allium sativum* L.) cloves, leek (*Allium ampeloprasum* var. porrum), Welsh onion (*Allium fistulosum*), and purple onion (*Allium cepa*). H_2_S emission was recorded using an H_2_S sensor gas analyzer portable device. For each plant, 300 g of fresh material was used.

As part of the characterization of H_2_S in these horticultural plants, the detection of H_2_S gas emission was performed using 300 g of cut materials. Figure 2 indicates that the species of the genus Allium were the only ones that allowed it to be detected. It is noteworthy that the emission of H_2_S is relatively quite fast and is maintained over time. Thus, leek reaches a maximum peak of 3.3 ppm of H_2_S after 30 min, followed by spring onion with a maximum peak of 7.4 ppm after 75 min, purple onion with 8.1 ppm after 90 min, and finally garlic with 9.0 after 180 min. It is noteworthy that the garlic maintained the emission of H_2_S up to 0.9 ppm after 21 h.

**Figure 3:**
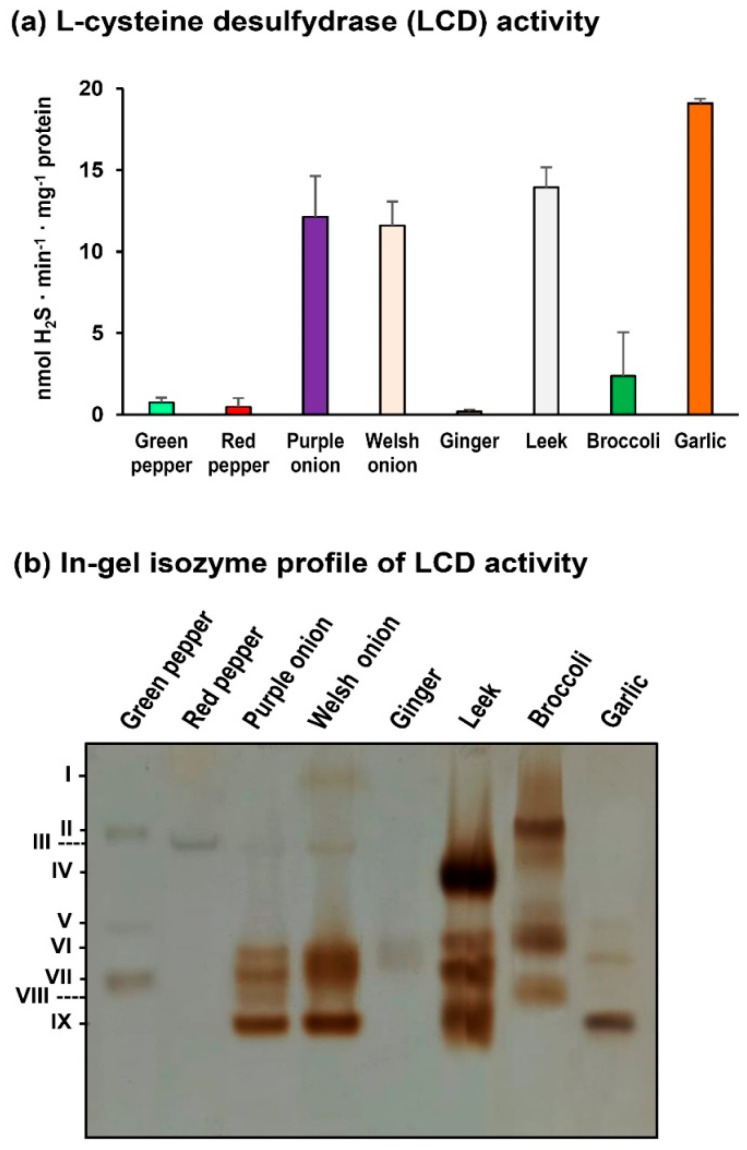
L-Cysteine desulfhydrase (LCD) activity in different plant samples. (**a**) Spectrophotometric assay. (**b**) In-gel isozyme profile of LCD activity. Protein samples (74 µg protein per lane) were separated by non-denaturing polyacrylamide gel electrophoresis (PAGE; 8% acrylamide) and the LCD activity was detected by lead acetate staining (see M&M for details).

Assuming that the L-cysteine desulfhydrase (LCD) activity is considered the enzyme that most contributes to the generation of H_2_S in the cell, its activity was measured spectrophotometrically and also in polyacrylamide gels under non-denaturing conditions. Figure 3a shows that species of the Allium genus have higher LCD activity, with garlic having the highest one, followed by broccoli, pepper, and ginger. On the other hand, Figure 3b shows the LCD isozymes profile in the different analyzed plant species. They were designated as I to IX according to their increasing electrophoretic mobility in the non-denaturing polyacrylamide gel. The number and relative abundance were quite different, while a single LCD isozyme is identified in ginger, three isozymes appear in garlic, broccoli, and green peppers, and up to four LCD isozymes in purple and Welsh onions. In pepper fruits, it is remarkable that the number of isozymes changes with ripening, having three in green pepper fruits and only one in red peppers.

## 3. Discussion

H_2_S is recognized as a key molecule with a signaling function in animal and plant cells, with similar regulatory properties to those exerted by nitric oxide (NO) in higher plants under physiological and stress conditions [9,33,34,35,36,37,38]. With the aim of obtaining a better understanding of this molecule in different plants with agronomic interest, the endogenous content, its emission, as well as its possible correlation with LCD activity have been comparatively studied, since it is considered the most relevant enzyme in the H_2_S production in plant cells.

One of the difficulties in determining the endogenous H_2_S content in a specific sample is based on its chemistry because, being a weak acid, it can be dissociated to hydrosulfide (HS^–^) and sulfide (S^2−^) anions in an aqueous solution according to the following equations: H_2_S(gas) ↔ H_2_S(aqueous solution) ↔ HS^−^ + H^+^ ↔ S^2−^ + 2H^+^. Other considerations that should be taken into account are the pH of the medium, the nature of biological samples, that H_2_S can interact with thiol groups present in peptides and proteins, the selectivity of the technical approach as well as the experimental conditions [2,15,18,39]. Otherwise, the development of new approaches such as the specific fluorescent probes has allowed the detection of H_2_S at the subcellular level by bioimaging approaches [40,41,42,43]; however, its quantification is still a challenge. In an earlier study in cucumber (*Cucumis sativus* L.) plants, an H_2_S concentration of 8 nmol · min^−1^ g^−1^ FW that was light-dependent [44] was reported and, after fumigation with SO_2_, the H_2_S content was 0.02 to 0.2 ng · g^−1^ dry weight [45]. In *Arabidopsis thaliana* and faba bean (*Vicia faba*) leaves, using a micro sulfide ion electrode, an H_2_S content between 1 to 5 μmol · L^−1^ was reported [46,47]. More recently, Jin et al. [48], using both methylene blue and electrode methods, determined the H_2_S content of 17 species in different developmental stages and different organs and they showed a wider range, from 0.177 to 0.708 μmol · g^−1^ FW, in which *Platycladus* sp. in the cypress family had the highest content whereas tobacco had the lowest content. Consequently, our data in the assayed horticultural plants are in good agreement with all these previous reports. Although the values of H_2_S content determined with the micro sulfide ion electrode provide relative measurements accurately and reliably, the difficulty to measure the H_2_S content when these values are low to 0.1 μmol · g^−1^ FW should be mentioned and, consequently, the obtained values should be interpreted with caution. 

The emission of volatile H_2_S is another aspect that could have great relevance in higher plants, although the available information is still very scarce. In an earlier study, Sekiya et al. [49], analyzed the H_2_S emission by gas chromatography in leaf discs incubated in the presence of 10 mM L-Cys from nine species (*Cucumis sativus*, *Cucurbita pepo*, *Nicotiana tabacum*, *Coleus blumei*, *Beta vulgaris*, *Phaseolus vulgaris*, *Medicago sativa*, *Hordeum vulgare*, and *Gossypium hirsutum*) and they found an emission around 40 pmol H_2_S · min^−1^ cm^−2^; however, the H_2_S emission was not observed in the presence of D-Cys. Rennenberg et al. [50], using a flame photometric sulfur analyzer, reported an H_2_S emission between 38 to 91 pmol H_2_S · min^−1^ cm^−2^ pumpkin leaf area. In our experimented conditions, we only observed H_2_S emission in the species of the genus Allium, and this emission was maintained in the time, particularly in garlic which was up to 9 ppm. It is well known that these species of the genus Allium have a characteristic smell and taste which is due to the sulfur-containing volatile flavor compounds. These volatile constituents are generated by the action of the enzyme alliinase, with a molecular mass between 13 and 35 kDa depending on the Allium species, when plant tissue is disrupted, the alliinase catalyzes the conversion of odorless S-alk(en)yl-L-cysteine sulfoxides (SACs), known as alliin, into volatile smelling thiosulfinates [51,52]. These SACs are synthesized from glutathione in the cytosol and when the compartmentalization of the alliinase, present in the vacuole, is broken down, it allows the metabolization of these SACs and its emission in volatile thiosulfinates [53,54,55]. In fact, these groups of sulfur-containing natural products present in the different species of Allium are correlated with the benefits associated with human health when they are part of our diet [52,56]. Thus, it is well documented that garlic consumption reduces some risk factors related to cardiovascular diseases such as high blood pressure, high cholesterol, platelet aggregation, blood coagulation, and the increased content of reactive oxygen species (ROS) [52,57,58,59,60]. The detected H_2_S emission in the different species of Allium is also well correlated with the beneficial effects exerted on human health [59]. In plants, these sulfur-containing molecules including H_2_S have great relevance in the resistance of crops against diverse fungal diseases [61,62].

L-cysteine desulfhydrase (LCD) catalyzes the desulfuration of L-Cys to generate H_2_S and is considered one of the main sources of this molecule in the cytosol of plant cells [63]. As was mentioned, biochemical and molecular approaches have revealed that LCD is implicated in diverse processes such as seed germination [64], root development [25,65], stomatal closure [26,27,66], drought tolerance [67], and fruit ripening [31,68]. 

In our experimental conditions, it is well correlated with the total LCD activity found in the Allium species with the highest level of endogenous H_2_S content as well as its emission. However, the available information about the number of LCD isozymes present in a specific tissue or plant species and its corresponding functions is to our knowledge very scarcer. Considering all analyzed species and tissues, the present data indicate the existence of nine LCD isozymes indicating the great diversity in the number and relative abundance which seems to support the differential potential physiological regulatory functions that they could have. For example, in the pepper fruit samples, it was observed that total LCD activity was downregulated during ripening from green to red fruits, which was well correlated with the diminishment of the LCD isozymes since two of them were not detected in red fruits. 

## 4. Materials and Methods

### 4.1. Plant Material

California-type sweet pepper (*Capsicum annuum* L., cv. Melchor) fruits were collected from plastic-covered experimental greenhouses (Syngenta Seeds, Ltd., El Ejido, Almería, Spain) whereas the other plants were acquired in the local market including broccoli (*Brassica oleracea* var. Itálica), ginger (*Zingiber officinale*) rhizome, fennel (*Foeniculum vulgare*), eggplant (*Solanum melongena*), leek (*Allium ampeloprasum* var. porrum), Welsh onion (*Allium fistulosum*), purple onion (*Allium cepa*) and garlic (*Allium sativum* L.) cloves. 

### 4.2. Preparation of Plant Extracts

Plant samples were ground to a fine powder in liquid N_2_ using an IKA^®^ A11 basic analytical mill. The resulting powder was suspended in 0.1 M Tris-HCl buffer, pH 7.5, containing 1 mM EDTA, 0.1% (*v*/*v*) Triton X-100, 10% (*v*/*v*) glycerol to a final plant material/buffer (*w*/*v*) ratio of 1:1 for pepper, fennel, and leek fruit; 1:2 for eggplant, garlic, ginger, and broccoli; and, 2:1 for red onion and Welsh onion. Homogenates were then filtered through two layers of Miracloth and centrifuged at 27,000× *g* for 20 min. The supernatants were used for subsequent analyses. For H_2_S endogenous quantification, the supernatants were mixed with antioxidant buffer (2 M NaOH, 170 mM sodium ascorbate, and 180 mM EDTA). In the case of enzymatic activity, the extraction buffer was similar but with a pH of 8.0. Protein content was determined by a standard Bradford assay using a reagent (Bio-Rad Laboratories, Hercules, CA, USA).

### 4.3. Endogenous H_2_S Quantification

H_2_S was measured in plant extracts for 5 min at 25 °C using a micro sulfide ion electrode (LIS-146AGSCM; Lazar Research Laboratories) attached to a voltage meter (Lazar Research Lab. Inc., Los Angeles, CA, USA, model ISM-146 H_2_S-XS). H_2_S concentrations were calculated from a standard curve made with sodium sulfide (Na_2_S) according to the micro-electrode manufacturer’s instructions.

### 4.4. H_2_S Gas Emission Hydrogen Sulfide Gas Detector 

H_2_S gas emission was recorded using a high accuracy H_2_S sensor gas analyzer portable device (NOBGP brand, model TK3Bcb4T78 model), which can measure H_2_S gas in a range between 0~100 μmol · mL^−1^, a resolution of 0.1 with an accuracy less than or equal to ±5% full scale. In all cases, 300 g of fresh plant samples were cut into homogeneous pieces and placed in a methacrylate hermetic box (10 mm-thickness walls): 25 (large) × 25 (width) × 30 (height) cm = 15.34 L, furnished with a lid made on the same material. The H_2_S gas detector was placed into the box and the H_2_S emission was recorded for 18 h. In the case of garlic, the cloves were used but for leek and Welsh onion, the stringy roots and dark green leaves were chopped off. 

### 4.5. Spectrophotometric Assay and In-Gel Isozyme Profile of L-Cysteine Desulfrydrase (LCD) Activity

L-cysteine desulfhydrase (LCD, E.C. 4.4.1.28) activity was spectrophotometrically determined by the release of H_2_S from L-Cys as described previously [69,70]. Briefly, the enzyme assay contained 1 mM L-Cys, 100 mM Tris-HCl, pH 8.0, 1 mM dithiothreitol, and plant extract in a final volume of 1 mL. After 15 min incubation at 37 °C, the reaction was stopped by the addition of 100 μL of 30 mM FeCl_3_ prepared in 1.2 N HCl and 100 μL of 20 mM N,N-dimethyl-p-phenylenediamine dihydrochloride prepared in 7.2 N HCl. The formation of methylene blue was measured at 670 nm, and the enzyme activity was calculated using the extinction coefficient of 15 × 10^6^ cm^2^ mol^−1^.

For in-gel isozyme profile analysis, protein samples were separated using non-denaturing polyacrylamide gel electrophoresis (PAGE) on 8% acrylamide gels. After the electrophoresis, the gels were incubated in the dark in a staining buffer containing Tris-HCl 100 mM, pH 7.5, L-cysteine 20 mM, lead acetate 0.4 mM, pyridoxal 5′-phosphate hydrate 50 µM and β-mercaptoethanol 20 mM until the appearance of brown bands [24,71].

## 5. Conclusions

H_2_S is a signaling molecule in both animal and plant cells. The analysis of its endogenous content as to its possible emission in higher plants to determine its physiological functions and in response to environmental stresses has been a challenge for years [72]. However, the information concerning H_2_S in horticultural species is still scarce; therefore, the present study provides new information on the content and emission of H_2_S in horticultural plants, particularly in the species of the Allium genus. This may be of great importance in horticultural crops, considering that H_2_S applied exogenously has been shown to exert multiple benefits for vegetables and fruits, since it has the capacity to preserve their quality during postharvest storage and prevents infections by pathogens because H_2_S stimulates phytohormone, reactive oxygen, and nitrogen metabolism [73,74,75,76,77,78]. Furthermore, the identification of different LCD isozymes in the analyzed horticultural species indicates the relevance of this enzyme in H_2_S metabolism and raises new questions about the specific function of each isozyme which could be modulated by environmental conditions or physiological processes such as was observed in the ripening of pepper fruits. Allium species have been recognized for a long time to have healthy properties [79], and among the sulfur compounds that it contains, H_2_S seems to be of great relevance [58,80]. On the other hand, considering the high H_2_S emission of Allium species, they should be regarded as a potential source of this gas for its possible biotechnological application in the horticulture industry since it can extend the quality of vegetables and fruits during postharvest storage.
